# Clinical photoacoustic/ultrasound dual-modal imaging: Current status and future trends

**DOI:** 10.3389/fphys.2022.1036621

**Published:** 2022-10-19

**Authors:** Yanting Wen, Dan Guo, Jing Zhang, Xiaotian Liu, Ting Liu, Lu Li, Shixie Jiang, Dan Wu, Huabei Jiang

**Affiliations:** ^1^ Department of Ultrasound Imaging, The Fifth People’s Hospital of Chengdu, Chengdu, China; ^2^ School of Computer Science and Technology, Chongqing University of Posts and Telecommunications, Chongqing, China; ^3^ Department of Psychiatry and Behavioral Sciences, Stanford University School of Medicine, Stanford, CA, United States; ^4^ Department of Medical Engineering, University of South Florida, Tampa, FL, United States

**Keywords:** photoacoustics, ultrasound, multimodal, review, imaging, clinical

## Abstract

Photoacoustic tomography (PAT) is an emerging biomedical imaging modality that combines optical and ultrasonic imaging, providing overlapping fields of view. This hybrid approach allows for a natural integration of PAT and ultrasound (US) imaging in a single platform. Due to the similarities in signal acquisition and processing, the combination of PAT and US imaging creates a new hybrid imaging for novel clinical applications. Over the recent years, particular attention is paid to the development of PAT/US dual-modal systems highlighting mutual benefits in clinical cases, with an aim of substantially improving the specificity and sensitivity for diagnosis of diseases. The demonstrated feasibility and accuracy in these efforts open an avenue of translating PAT/US imaging to practical clinical applications. In this review, the current PAT/US dual-modal imaging systems are discussed in detail, and their promising clinical applications are presented and compared systematically. Finally, this review describes the potential impacts of these combined systems in the coming future.

## Introduction

Photoacoustic tomography (PAT) is an emerging method that provides a sub-millimeter spatial resolution image with a penetration depth of several centimeters, which is achieved by the combination of optical excitation and acoustic detection (Beard, 2011; Dai et al., 2017a; Yang and Ghim, 2021). Photoacoustic effect is induced by a nanosecond pulsed laser source. With the light illuminating the targeted tissue, it is absorbed by molecules inside the targeted tissues, leading to a temperature rise and thermoelastic expansion. In response, with the generated broadband acoustic waves, the signals are subsequently detected by conventional ultrasound (US) transducers. Multispectral optoacoustic tomography (MSOT) is based on the principle of PAT, which distinguishes absorbers based on their spectral signatures, due to various optical absorbers such as endogenous material (hemoglobin, melanin, lipids, water, and other chromophores in human) or exogenous contrast agents (Ntziachristos and Razansky, 2010; Bayer et al., 2013; Li M et al., 2018).

Over the recent years, the number of studies on PAT systems in the literature has been increasing rapidly (Su et al., 2010; Beard, 2011; Menke, 2015; Wang et al., 2016; Upputuri and Pramanik, 2017; Dong et al., 2017; Choi et al., 2018; Jo et al., 2018; Li X et al., 2018; Lin et al., 2018; Manohar and Dantuma, 2019; Nyayapathi and Xia, 2019; Steinberg et al., 2019; Zhao et al., 2019; Duan et al., 2020; Manohar and Gambhiret, 2020; Deán-Ben and Razanskyet, 2021; Gröhl et al., 2021; Li et al., 2021; Wen Y. T. et al., 2022; Yang and Ghim, 2021; Wen YT. et al., 2022). In these studies, the system designs have in common that an optimized imaging system achieved a higher spatial and temporal resolution, better penetration in tissue with reduced artifacts. Consequently, the advancements in PAT have enabled a wide applications ranging from small animal studies to clinical imaging, including imaging of breast (Becker et al., 2018; Xu et al., 2019; Yang et al., 2020), thyroid (Dima and Ntziachristos, 2016; Sinha et al., 2017; Roll et al., 2019), skin (Petri et al., 2016; Dahlstrand et al., 2020), tumors (Li et al., 2015; Yamada et al., 2020; Karmacharya et al., 2021; Knorring and Mogensen, 2021; Wang C et al., 2021), cardiovascular (Taruttis et al., 2013; Karlas et al., 2021a), functional neuroimaging (Wang et al., 2003; Wu et al., 2019a), eyes (Liu and Zhang, 2016) and others (Nagae et al., 2018; Yang et al., 2018; Liang et al., 2021; Yan et al., 2021). Therefore, PAT imaging has broader clinical translational potential than other forms of pure optical imaging, indicating its ability to provide potent structural, functional, and molecular information *in vivo* (Yao and Wang, 2018; Wu M et al., 2021). Notably, although the PAT image shows the heterogenous localization in tumors, combining US with these images can provide the exact anatomical co-localization and establish suspect region of interest (ROI), which allows for a more detailed PAT analysis of these ROIs (Manohar and Dantuma, 2019; Park et al., 2017a). Hence, it suggests the necessity of combining PAT with another structural imaging modality with deep tissue penetration to achieve feasible clinical applications.

Ultrasound, as a common imaging technique with deep tissue penetration, high spatial resolution, and properties of real-time imaging, has been widely used in clinics (Bene et al., 2022; Paola et al., 2022). Nonetheless, its non-whole body imaging, poor osseous and gas-containing organ penetration capabilities limit further development. In response to these issues, various multi-modal imaging systems have been integrated to provide complementary information, which consequently boost the sensitivity and specificity for disease diagnostics.

Since the hybrid nature of PAT makes it easy to integrate with the existing US imaging systems (Jennings and Long, 2009), several studies have been conducted on PAT/US dual-modal systems. As an obvious reason for this combination, it allows for the use of the same piezoelectric transducer, as well as the same data acquisition (DAQ) process for PAT/US signal detection (Das et al., 2009; Bouchard et al., 2014). Consequently, the data for both images is acquired simultaneously and gathered. Additionally, morphologic information produced by US imaging, including tissue or lesion boundaries (due to different sound speed and acoustic impedance) helps PAT image reconstruction and facilitates its semi-quantitative or quantitative assessment by tissue functional/molecular parameters (Beard, 2011; Dong et al., 2017).

In the last two decades, several studies on PAT/US imaging in phantom and animals were reported (Aguirre et al., 2009; Nam et al., 2012; Kruizinga et al., 2013; Karpiouk et al., 2012; Gerling et al., 2014; Luke et al., 2014; Luke and Emelianov, 2015; Mallidi et al., 2015a; Mallidi et al., 2015b; Raes et al., 2016; Salehi et al., 2016; Kang et al., 2017; Yamaleyeva et al., 2018; Dumani et al., 2019; Hartman et al., 2019; Dadkhah and Jiao, 2021; Kang et al., 2022). Moreover, by combining PAT with US imaging, it has the potential to obtain accurate and clinically relevant imaging data for diagnostic and therapy-monitoring purposes (Mallidi et al., 2015b). Several comprehensive researches for preclinical/clinical applications on PAT/US systems, such as PAT/US for healthy tissue (Lou et al., 2017; Kim and Chang, 2018; Mennes et al., 2018), tumors and metastasis (Garcia-Uribe et al., 2015; Neuschler et al., 2017; Yang et al., 2017; Becker et al., 2018; Li M et al., 2018; Shiina et al., 2018; Xavierselvan et al., 2021), bones and joints (van den Berg et al., 2017; Feng et al., 2020) and cardiovascular (Kruizinga et al., 2013; Karlas et al., 2021a; Karlas et al., 2021b; Wu M et al., 2021; Wu Y et al., 2021) were reported.

In addition, multifunctional contrast agents for PAT and US imaging were synthesized decades ago (Kim et al., 2010a). To date, as there are still barriers for clinical use of dual-modal contrast agents, most studies have been focused on the preclinical stage so far. Potentially, simultaneous PAT/US imaging enhanced by contrast agents such as microbubbles or nanobubbles can be a valuable tool for intraoperative assessment of tumor boundaries and resection margins. Thus, it will open a novel avenue of translating PAT/US imaging to clinical applications. Furthermore, given the nonionizing radiation, wide availability, portability, and low-cost nature of PAT/US imaging, this technology is thusly unique when combined with photodynamic therapy (PDT), providing multiparametric anatomical and functional information in therapeutic process. In addition, due to its label-free nature, PAT/US is capable of long-term longitudinal monitoring for image-guided treatment.

This review will focus on the progress of PAT/US systems with a potential of being translated into clinical applications. The review is organized as following: in Section 2 we discuss the overall comparison of technological advances in translational clinical PAT/US systems; in Section 3 is dedicated to PAT/US systems towards various clinical applications; in Section 4; we will briefly conclude and discuss the future directions of PAT/US for clinical translations.

## PAT/US system for clinical translation

Development of PAT/US imaging systems has been proceeded in all aspects, including hardware, instrument design, and image reconstruction algorithms (Poudel et al., 2019; Tian et al., 2020). In this section, we present feasible dual-modal platforms from a perspective of radiologists and clinicians.

### Graphical user interface

In order to achieve clinical utility of an imaging system, a friendly GUI of the platform is important. In recent decades, the integration technologies of PAT/US platform with friendly GUI have been extensively studied (Aguirre et al., 2009; Gerling et al., 2014; Dumani et al., 2019; Kang et al., 2022). So far, the combination of PAT and US imaging system in clinical translation has been demonstrated by different research groups (Niederhauser et al., 2005; Kolkman et al., 2006; Wang et al., 2011; Neuschler et al., 2017; Kim et al., 2020; Han et al., 2022). User-friendly operation is a critical requirement for successful clinical translation of a PAT/US system, such that radiologists or clinicians can use the system by themselves.

The first generation PAT/US system utilizes a duplex PAT imaging device with a single handheld duplex probe (Jennings and Long, 2009; Neuschler et al., 2017). The main feature of these devices are a programmable software, which allows one to modify the operation sequence of the system. There are two modes of GUI on the study devices: 1) US mode, generating only grayscale US images, and 2) PAT/US mode, generating grayscale US images fused with functional PAT data. In this way, the received PAT signals are coregistered with grayscale US signals, generating real-time and pseudo-colored maps of relative oxygenated hemoglobin (HbO_2_) and deoxygenated (HbR). Accordingly, total hemoglobin (HbT) concentration can be calculated through the system. However, they suffered from the limitation of lack of real-time modification of the imaging parameters by users, hence the user-friendly GUI needs to be explored in further study to help the user to optimize the images.

A system having capability to support real-time parameter modification during imaging was designed, which can optimize the images by using the parameter control software, including time gain compensation coefficients adjustment, dynamic range for image optimization, the frequencies (center frequency, cutoff frequency, and filter size) demodulation and the beamforming options (beamforming method, apodization window type, and speed of sound) modification. Accordingly, the users could optimize the images in real-time by a MATLAB-based software without pausing the imaging operation during the image acquisition process (Kim et al., 2020). This system advances PAT/US device one more step closer towards the translation clinical use.

### Design of PAT/US probes

**FIGURE 1:**
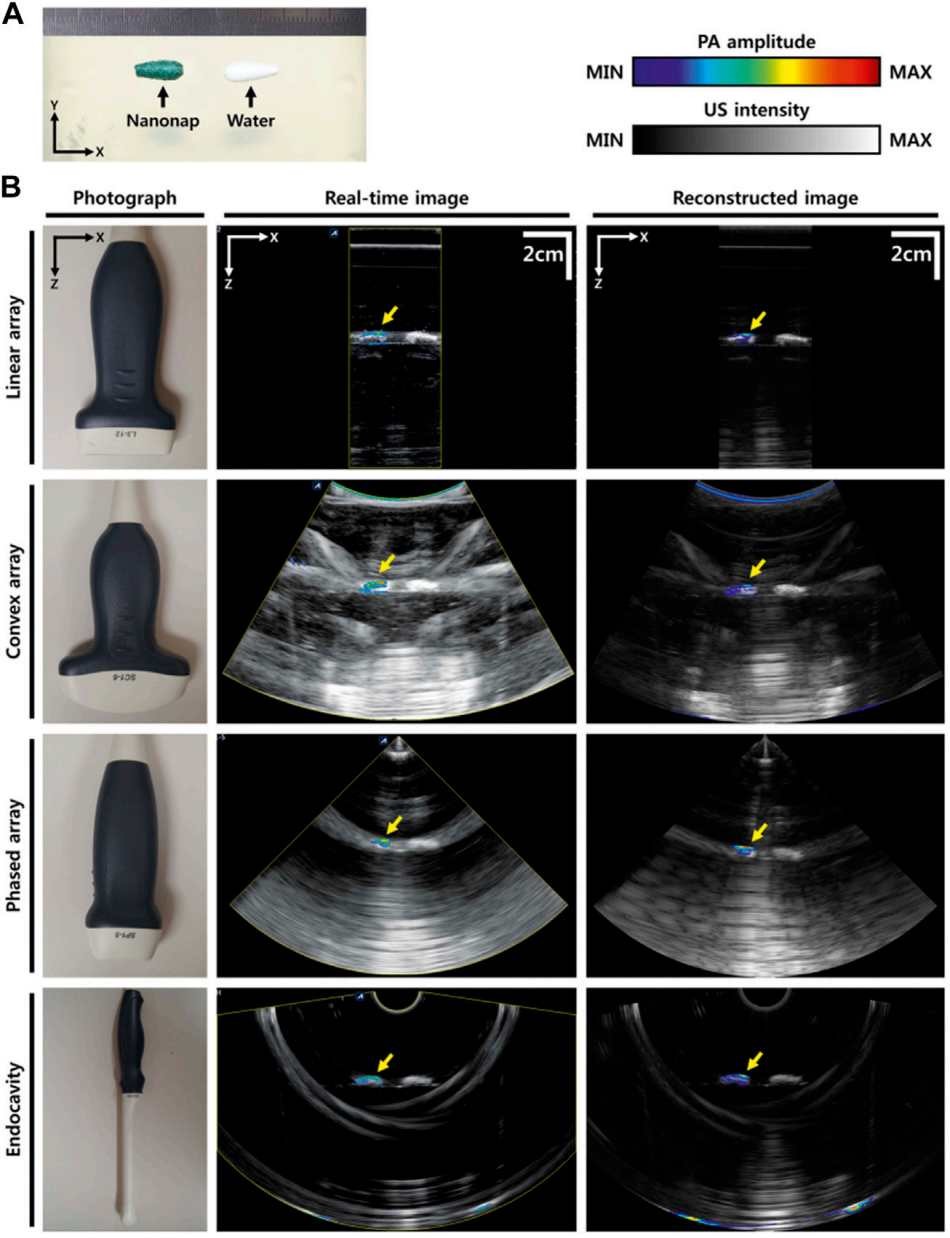
PAT/US images using different transducers. **(A)** Photograph of the phantom. **(B)** Photographs of different transducers (linear array, convex array, phased array, and intracavity transducers), real-time PAT/US images, and reconstructed PAT/US images acquired by these transducers. PAT, photoacoustic tomography; US, ultrasound. Reprinted with permission from [Kim J., Park S., Jung Y., Chang S., Park J., Zhang Y., et al. (2016). Programmable Real-time Clinical Photoacoustic and Ultrasound Imaging System. Sci Rep.6,35137. doi: 10.1038/srep35].

Various research groups have implemented integrated PAT and US probes for clinical applications (Levi et al., 2014; Dai et al., 2015; Kim et al., 2016; Miranda et al., 2018; Kothapalli et al., 2019; Zhang and Wang, 2020; Pang et al., 2022). A programmable US system can utilize various US transducers (Figure 1) to generate PAT/US images (Kim et al., 2016). In order to acquire images of different depth of human *in vivo*, the PAT/US platform is fully compatible with several different probes. A novel clinical PAT/US system with various US probes were designed as follows: 1) A linear array (L3-12); 2) A convex array (SC1-6); 3) A phased array (SP1-4); and 4) An intracavity transducer (EC3-10H). Accordingly, the PAT/US system can be applied to a wide range of clinical applications by selecting suitable sensors, from superficial tissues to deep organs at different depths in human.

The design of the integrated PAT/US imaging probe is also updated for efficient light delivery and optimized the geometry of the imaging probe. An arc-array was established based on commercial US systems (iThera Medical, Germany) (Levi et al., 2014), which was suitable for small organs detection *in vivo*. Due to the relatively small field of view (FOV) of the arc-array transducer, it might not be suitable for the large region. Meanwhile, the linear-array transducers were developed for preclinical and clinical trials, including iU22 (Philips Healthcare) (Garcia-Uribe et al., 2015) and EC-12R (Alpinion Medical Systems, Republic of Korea) (Kim et al., 2016). However, these high-frequency transducers suffered from a shallow imaging depth, which were not suitable for deep organ detection, thus were limited for general clinical applications.

The developed system can be used to provide new biological information in diagnostic fields such as uterus, bowels, vascular and organs adjacent to cavity viscera (prostate, pancreas, etc.). The endoscopic image modalities have been proposed (Dai et al., 2017b; Li et al., 2019). Emerging endoscopy techniques refers to PAT is photoacoustic endoscopy (PAE), which incorporates PAT in a small probe to visualize internal organs through intra-cavity introduction (Wang, 2008). In addition to PAE, researchers are exploring a PAT/US “mini-probe” integrated into a conventional endoscope’s instrument channel, which is a promising strategy to achieve clinical benefits (Yang et al., 2012). In this study, a simultaneous PAE/EUS dual-modal system with a streamlined shape probe (3.8-mm-diameter) was designed to image internal organs *in vivo* (Yang et al., 2012). However, it could not provide a 360° field of vision which is often needed in clinical settings. Later, a miniaturized PAE/EUS system with a 2.5-mm-diameter probe was conducted (Li X et al., 2018). This catheter provided a full (360°) field-of-view cross-section images, which was comparable with the 2.8-mm instrument channel of conventional clinical endoscopes. A novel intrauterine PAT/US imaging probe (linear array, 15-MHz) was designed to detect endometrial diseases *in vivo* (Miranda et al., 2018). For application within the human uterus, the intra-cavity probe (2-mm-diameter) is comparable with an endometrial suction curette, which is a catheter-like device with a diameter of <3 mm. As a custom designed probe for deep tissue, the imaging depths up to several centimeters have been achieved. A miniaturized capacitive micromachined ultrasonic transducer (CMUT) array for simultaneous imaging of transrectal PAT/US system were combined for human prostate detection *in vivo* (Kothapalli et al., 2019).

Moreover, with the system equipped with a three-dimensional (3D) detection aperture, the spiral 3D images were reconstructed for PAT/US images. Compared to traditional sensors, this transducer had a higher bandwidth, and the signal-to-noise ratio (SNR) was improved as well. It has the potential to be introduced in clinics in the future. As well, current acoustic-resolution PAE/USE generally employs a point-focused transducer which is only sensitive in its focal region. As a result, the sensitivity and lateral resolution dramatically reduce when the targets move out of its focus. A designed line-focused transducer emits a more uniform sound field, as compared to a point-focused transducer, resulting in the original signal intensity and SNR of the adjacent targets to be closer in the radial direction, which improves the uniformity of target signals in hybrid imaging (Pang et al., 2022). Further study is need to evaluate the diagnostic ability and accuracy of abovementioned PAE/USE transducers in larger clinical trials.

### Miniaturization of PAT/US device

In order to achieve certain clinical applications, miniaturized PAT/US imaging system were designed by several groups (Kothapalli et al., 2019; Kim et al., 2020). For instance, a devised portable PAT/US system which can visualize vascular distribution without injecting any contrast agent was designed (Kim et al., 2020). While US and certain optical technologies are available in the size of a mobile phone (Ahn et al., 2015), PAT/US systems still need to be improved to reach that level of portability, mainly focusing on advanced laser source and high performance DAQ system.

**FIGURE 2:**
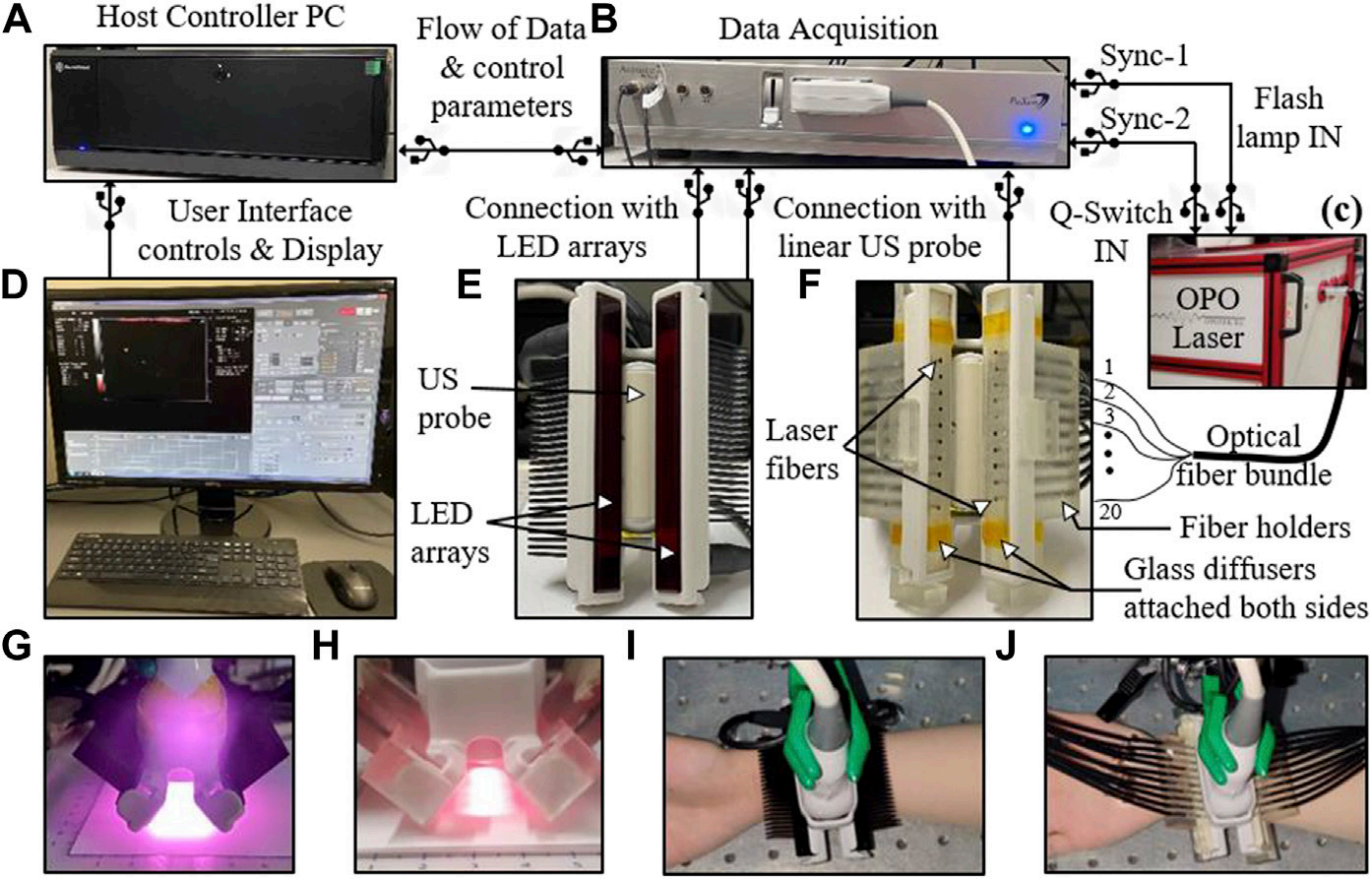
The experimental setup designed for comparing light-emitting diode (LED)-based and high-power laser-based PAT and US imaging. **(A)** A host controller PC; **(B)** DAQ hardware; **(C)** A portable high-power laser with its output coupled to the input end of an optical fiber bundle; **(D)** Computer display: displays PAT (red scale), B- mode US (grayscale), and coregistered PAT/US (overlaid red PAT on gray US); **(E)** Arrangement of two 850 nm LED arrays around the US probe; **(F)** Arrangement of twenty laser fibers inserted into the two fiber holders around the US probe; **(G,H)** Optical illumination profile achieved with two LED array sources and laser sources, respectively; **(I,J)** Pictures of a human wrist under imaging with the LED and laser arrangements, respectively. LED: light-emitting diode; PAT, photoacoustic tomography; US, ultrasound; DAQ: data acquisition; PC: personal computer. Reprinted with permission from [Agrawal S., Singh M. K. A., Johnstonbaugh K., Han D. C., Pameijer C. A., and Kothapalli S-R. (2021). Photoacoustic Imaging of Human Vasculature Using LED versus Laser Illumination: A Comparison Study on Tissue Phantoms and *In vivo* Humans. Sensors. 21,424. doi: 10.3390/s21020424].

Conventionally, a high-power laser source is employed for most dual-modal PAT/US systems (Kothapalli et al., 2019; Regensburger et al., 2019; Agrawal et al., 2021a). In order to translate PAT/US technology to clinical applications, especially for point of care (POC) detection, a significant reduction in both cost and size of laser source is required. To address this issue, several cost-effective alternatives for the optical excitation/detection (Liu and Zhang, 2016; Upputuri and Pramanik, 2017 have been explored. Recently, the light-emitting diodes (LEDs) have emerged as portable optical sources for PAT *in vivo* (Anas et al., 2018; Jo et al., 2018; Xia et al., 2019; Farnia et al., 2020; Maneas et al., 2020). However, these state-of-art LED arrays carry significantly lower optical energy (<0.5 mJ/pulse) and high pulse repetition frequencies (PRF) (4 KHz) compared to the high-power laser sources (100 mJ/pulse) with low PRFs of 10 Hz. To enhance the performance of LEDs, an arrayed arrangement of LED elements was developed (Zhu et al., 2018; Zhu et al., 2020), thereby increasing the pulse energies from a few µJ to hundreds of µJ. In addition, higher PRF of the LED allowed a sufficient and fast PAT signal averaging which led to significant SNR improvements for deep tissue imaging. A commercial LED based PAT/US system (Figure 2) was designed (Agrawal et al., 2021b) with a lower mean noise compared to the laser based PAT/US system. However, the SNR value for the laser-based PAT image was about 6 dB lower than the SNR with the LED array acquisition. Therefore, due to the low power of LED arrays, a higher frame averaging is required to image deep tissue targets. Nevertheless, LED-PAT/US systems have strong potential to be a mobile health care technology for clinical applications.

### Toward clinical translation PAT/US device

In order to achieve highly translatable to the clinical field, several PAT/US systems based on commercial US system were conducted without the need of additional hardware and algorithms to obtain data and reconstruct images, making it compatible with most commercial US platforms (Park et al., 2021). However, direct use of a US commercial system to acquire quality PAT image is difficult. The most challenging issue is how to recover weak PAT signals from expected subsurface tissues, since the signal level in US are generally much higher than those in PAT, and neither the SNR nor the digitizer threshold of most commercial US systems are suitable for directly PAT imaging.

At the initial stage towards a clinical PAT system, several commercial PAT/US platforms have been used in preclinical applications, including the iU22, Phillips Healthcare, Netherlands (Kim et al., 2010b), the VevoLAZR series (FujiFilm VisualSonics, Canada) (Needles et al., 2013), the MSOT Acuity series (iThera Medical, Germany) (Levi et al., 2014) and the Vintage series (Verasonics, United States) (Kothapalli et al., 2019). However, for general clinical research, these systems suffered from several limitations, such as immobile laser, unprogrammable US machine and without approvement of the United States Food and Drug Administration (FDA). Later, in order to overcome the limitations, a clinically applicable PAT/US system was developed (Kim et al., 2015). A portable pulsed laser (Phocus, OPOTEK, United States) and a FDA-approved clinical US machine (EC-12R, Alpinion Medical Systems, Republic of Korea) were integrated in the system. However, the abovementioned systems only combine PAT with US for imaging, without utilizing other optical imaging modalities such as photoacoustic microendoscopy (PAM) imaging, which providing microvascular networks map in superficial tissue. Consequently, a linear transducer combined PAT, PAM, and B-mode US imaging into one commercial US platform was design (Wang S. L et al., 2021). As compared to existing multi-modality systems based on PAT and US, this system provides more complementary morphological and functional information of tissue *in vivo*. It has potential to achieve the best benefits of integrated PAT/US and promised for multi-scale and multi-functional imaging for clinical applications in the future.

## Clinical applications of PAT/US system

For clinicians or radiologists, “seeing is believing”. In the last several years, several clinical studies on PAT/US dual-modality were reported, including human thyroid (Dima and Ntziachristos, 2016; Yang et al., 2017; Kim et al., 2021), breast (Garcia-Uribe et al., 2015; Becker et al., 2018; Neuschler et al., 2017; Nyayapathi and Xia, 2019; Kelly et al., 2020; Goh et al., 2019; Yang et al., 2020), skin (Oraevsky et al., 2018; Park et al., 2021), extremities (Xia et al., 2015; Mercep et al., 2015; Liu and Zhang, 2016; Kim et al., 2016; Jo et al., 2017; Oeri et al., 2017; van den Berg et al., 2017; Feng et al., 2020; Daoudi et al., 2021), prostate (Agrawal et al., 2020; Kothapalli et al., 2019), bowels (Knieling et al., 2017; Leng et al., 2018; Yang et al., 2019), vascular (Karpiouk et al., 2012; Wu et al., 2015; Andrei et al., 2021), placenta (Xia et al., 2015; Maneas et al., 2020) and others (Jose et al., 2009; Gonzalez et al., 2021; Mozaffarzadeh et al., 2021). With the combination of the two modalities in one imaging system, it is acceptable for radiologists or clinicians to adapt and associate morphological features with functional information. On the other hand, US detection is naturally registered with PAT detection; therefore, researches using US-guided PAT detection were included in this review. We compared the properties of each dual-modality system from device performance to clinical trials, and fully discuss their advantages and disadvantages, as well as possible clinical applications in future.

### Thyroid imaging

Thyroid tumors are common tumors in the head and neck. Newly-diagnosed thyroid cancer cases have increased due to advancements in diagnostic imaging techniques such as US, X-rays, and magnetic resonance imaging (MRI) (Vaccarella et al., 2016). Although the incidence of malignant cases of all the discovered thyroid nodules were about 10%, not all nodules need to be treated immediately. Hence, it is estimated that over 560,000 patients were overdiagnosed over the last two decades (Ahn et al., 2014; Vaccarella et al., 2016). The American Thyroid Association recommended US as a routine thyroid examination for all patients with thyroid lesions and for healthy people (Gharib et al., 2016). However, conventional color Doppler US has limited capacity in discriminating untypical benign and malignant nodules. An accurate diagnosis of thyroid disease can be aided by reliable vascular information. In recent years, contrast-enhanced ultrasound (CEUS) has been applied for the clinical evaluation of the thyroid nodule. However, CEUS is rather invasive for the intravenous injection of contrast agent. Therefore, a noninvasive functional imaging modality, with the ability of evaluating the morphological and functional information simultaneously, will be beneficial to the early diagnosis and clinical management of thyroid tumors. PAT is a novel hybrid imaging modality, which relies on sensitive tissue optical properties. Therefore, PAT can provide important functional information, such as the oxyhemoglobin saturation (SO_2_).

**FIGURE 3:**
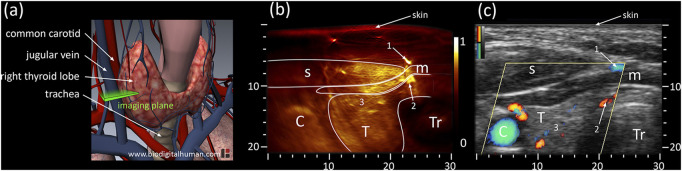
**(A)** Anatomy of thyroid including cardio-vascular and respiratory system; the cross-sectional imaging plane is highlighted in green. **(B)** PAT and **(C)** US cross-sections of the left thyroid lobe of the first volunteer. PAT: Photoacoustic tomography, US: Ultrasound; C: Carotid T: Thyroid, Tr: Trachea, s: sternocleidomastoid muscle, m: infrahyoid muscle; axes in mm. Reprinted with permission from [Dima A., Ntziachristos V. (2016). *In-vivo* handheld optoacoustic tomography of the human thyroid[J]. Photoacoustics, 4(2):65–69. doi: 10.1016/j.pacs.2016.05.003].

PAT combined with US can provide important clue in the diagnosis of thyroid disease with reliable vascular information in an initial clinical study (Banaka et al., 2011). Then some thyroid nodules studies have been conducted using costumed PAT/US system (Dima and Ntziachristos, 2016; Yang et al., 2017; Kim et al., 2021). A curved US array (Dima and Ntziachristos, 2016) and linear array (Yang et al., 2017) were employed to delivery high-fidelity performance in human thyroid *in vivo*, respectively. Both studies demonstrated that it was possible to detect the thyroid’s outline and identify vascular features. In addition, it was found that PAT was more efficient at detecting blood vessels compared to colored Doppler US (Figure 3). Later, multi-spectral acquisition was used to further improve accuracy by tissue oxygenation parameter in thyroid imaging (Kim et al., 2021). All the single parameter analyses showed encouraging results with statistically differentiable distributions. Furthermore, they successfully visualized real-time PAT/US images of thyroid nodules.

Taken together, previous clinical trials have demonstrated that PAT with a high-quality clinical US system can provide high-quality morphological and functional images in human thyroid *in vivo*. Subsequent developments are needed to further update the dual-modal system in several regards: 1) A large FOV is required for efficient navigation during monitoring and needle guidance; 2) improving the duplex probe for easier hand-held operation; 3) To improve PAT SNR, optimized laser delivery is needed to achieve higher spatial resolution and less artifacts; 4) Motion artifact causing by the target and operator movement between multiple laser pulses; 5) Analyses of MSOT parameters and comparisons between different types of thyroid cancer needs further study; 6) Multispectral parameters processed and displayed in GUI in real time.

### Breast imaging

In 2022, over 110,000 new cases of breast cancer will be diagnosed in the United States, making it the most common cancer for women worldwide (Porter, 2009; Ghoncheh et al., 2016; Siegel et al., 2021). Conventional breast imaging techniques, like X-ray mammography and US, primarily focus on morphological changes of breast tissue to discriminate benign from malignant tissue. X-ray mammography is not reliable for women with dense breasts with ionizing radiation and US is strongly operator dependent (Heijblom et al., 2012). The hybrid nature of PAT breast imaging provides both structural information and hemoglobin-related functional information within the breast, which can aid clinical diagnosis. In addition, since breasts have protruding geometry in the superficial region, they are optically transparent compared to other organs, making them ideal to image with PAT. Several clinical studies indicate that angiogenesis begins at an early stage of breast carcinoma *in situ*, with this understanding, the dual-modal PAT/US imaging based on two different contrast mechanisms (functional optical and anatomical US) can achieve greater clinical performance with a merit of radiation-free, breast-compression-free, and relatively inexpensive.

**FIGURE 4:**
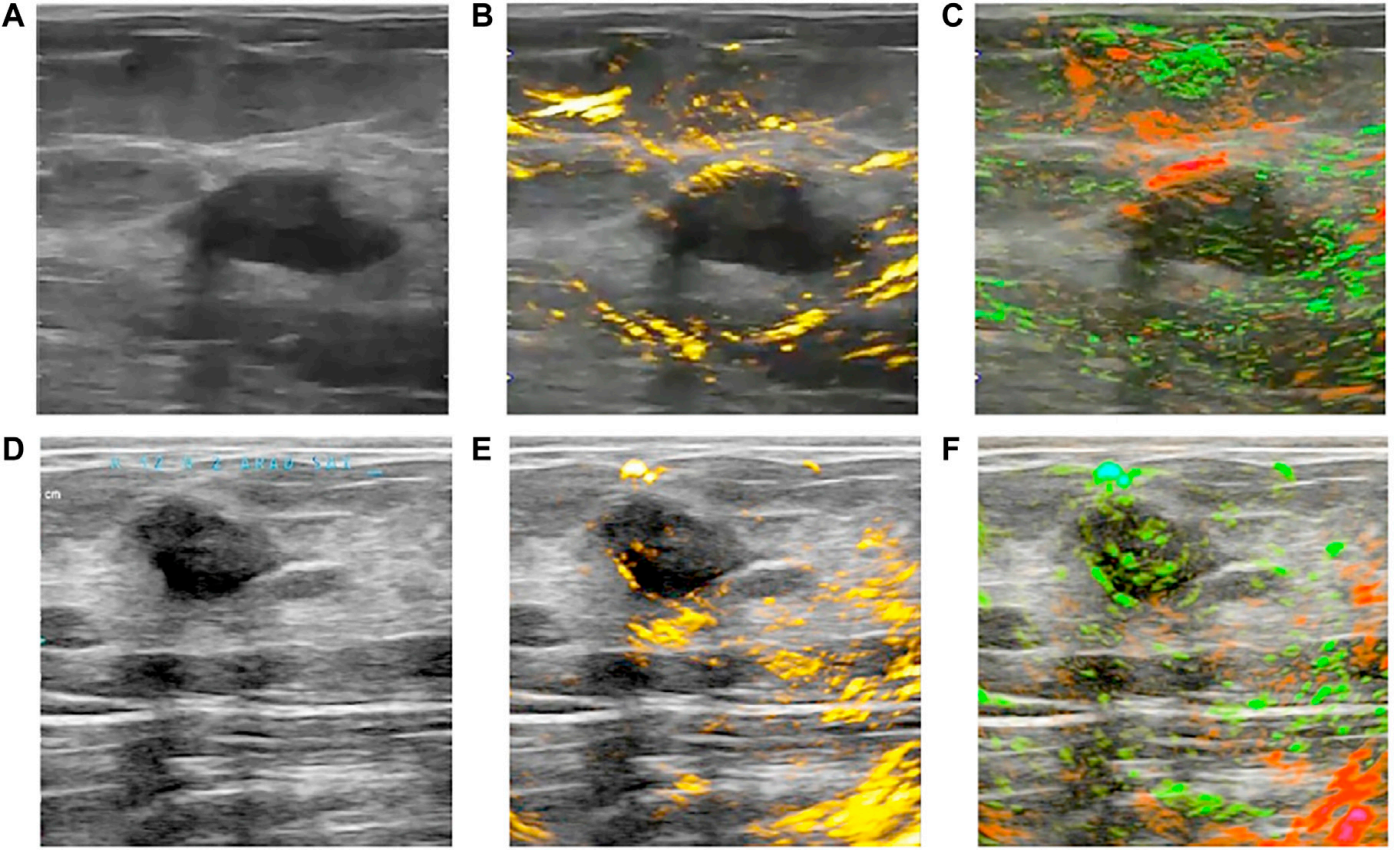
Clinical OA/US imaging of two benign breast tumors. **(A,D)** show US of two breast tumors; **(B)** reveals a lack of angiogenesis microvasculature within the breast tumor in OA/US image; **(C)** shows the majority of the tumor is normally oxygenated in OA/US image; **(E, F)** display another similar breast tumor in OA/US image. OA: optoacoustic; US: ultrasound. Reprinted with permission from Oraevsky et al. (2018).

In recent years, combined PAT/US technology has demonstrated its clinical feasibility in human breast cancer diagnosis *in vivo* (Becker et al., 2018; Oraevsky et al., 2018; Han et al., 2022) and (Goh et al., 2019) *ex vivo* in humans. Advanced systems explored for real-time PAT/US breast imaging with high temporal feature have been designed (Becker et al., 2018; Oraevsky et al., 2018). The individual images are reconstructed at possible rate of 25 Hz for single wavelength imaging and the MSOT image is possible at a 5 Hz refresh rate per multispectral image (Becker et al., 2018). MSOT/US image was conducted to acquire functional information of breast cancer, revealing increased signals for HbR and HbO_2_ in invasive breast carcinoma (Figure 4). Another real-time PAT/US system named Imagio™ was developed for testing in a multicenter clinical trial termed PIONEER (Oraevsky et al., 2018). A spatial-temporal coregistration of functional and anatomical images is explored in clinical trials (Figure 4). In this system, radiologists can evaluate the vascular pattern around tumors, the microvascular density of lesions, and the relative values of HbT, SO_2_ to adjacent tissues. While the Imagio™ lacks the advantages of US, such as speed of sound and acoustic attenuation measurement, further study would focus on improvement of both PAT and US performance on this system (Zalev et al., 2012; Oraevsky et al., 2018; Stephens et al., 2021).

Large sample study and data set for PAT/US system is as a bridge from trials to clinical applications. Analyses in two large samples of PAT/US breast imaging were performed (Neuschler et al., 2017; Kim et al., 2020), enrolling 2105 women and 2000 patients, respectively. A comparison on Breast Imaging Reporting and Data System (BI-RADS) categories using PAT/US versus US alone was completed (Neuschler et al., 2017). With a similar sensitivity (US: 96% vs. PAT/US: 98.6%), the specificity of PAT/US exceeded that of US by 14.9%. While PAT will not replace any US functions, but provides important complementary information for US imaging. The result demonstrated it may be possible to improve the specificity of breast mass assessment by using PAT/US, thereby reducing the number of false-positive examinations and biopsies. However, it also had limitation that led to some false-negative interpretations. Later, another multicenter clinical trials demonstrated the clinical feasibility with a hand-held duplex technology (Kim et al., 2020). Complex signal processing and image reconstruction algorithms in the software enable real-time coregistration of PAT/US imaging.

In comparison to two-dimensional (2D) functional PAT/US imaging, 3D functional PAT/US imaging has several advantages. Using 3D PAT/US imaging can provide quantified results based on 2D PAT imaging, which can better represent the overall functional imaging features of breast tumor. To verify this, Yang et al. explored a quantitative method to analyze characteristics of breast tumors using 3D volumetric data obtained from a 3D PAT/US functional imaging system (Yang et al., 2020). The analysis of the 3D distribution of vessels could provide a more comprehensive description of the tumor vasculature than 2D analysis. Furthermore, 3D quantification of PAT functional information may be able to minimize intra-observer differences compared to previous 2D PAT/US imaging studies. Limitations of this research include the “limited view” problem, causing most of the reconstructed vessels to have an orientation that tended to be parallel with the scanning direction.

Taken together, PAT/US imaging has provided meaningful information for a radiologist to accurately diagnose malignancy. Notably, for patients with breast cancer, PAT/US is accelerating its clinical translation in macroscopic and microscopic imaging. To define the role of PAT/US in clinical practice, further research should be conducted into feature analysis and interpretation strategies. Moreover, further technical advances of the technology will be envisioned in the direction of quantitatively accurate PAT/US image and the 3D PAT/US systems with large FOV for human breast.

### Skin imaging

Skin cancer is one of the most common types of cancers affecting around one out of five people in most developed countries (Deán-Ben and Razanskyet, 2021). The most aggressive type of skin tumors is malignant melanomas. The melanoma patients have a very poor prognosis if it is not identified and treated in early stage accurately. Recently, noninvasive imaging techniques, such as high-frequency ultrasonography (HFUS), reflectance confocal microscopy (RCM), and optical coherence tomography (OCT) have been developed to improve the diagnostic sensitivity and accuracy for skin melanoma (Heibel et al., 2020). However, those methods are neither sufficient to measure the accurate depth of the melanoma nor accurately estimate the real invasive depth of the tumor (Crisan et al., 2013). PAT image is gaining great attention as a noninvasive and nonionizing diagnostic method to visualize skin melanomas, due to the presence of strong melanin contrast in tumor. The spectral PAT images were also integrated to a pulse-echo US image serving as anatomical reference. In the last couple of years, several groups have conducted experiments of skin melanoma in animals to confirm its feasibility using PAT imaging system (Neuschmelting et al., 2016; Hindelang et al., 2019).

**FIGURE 5:**
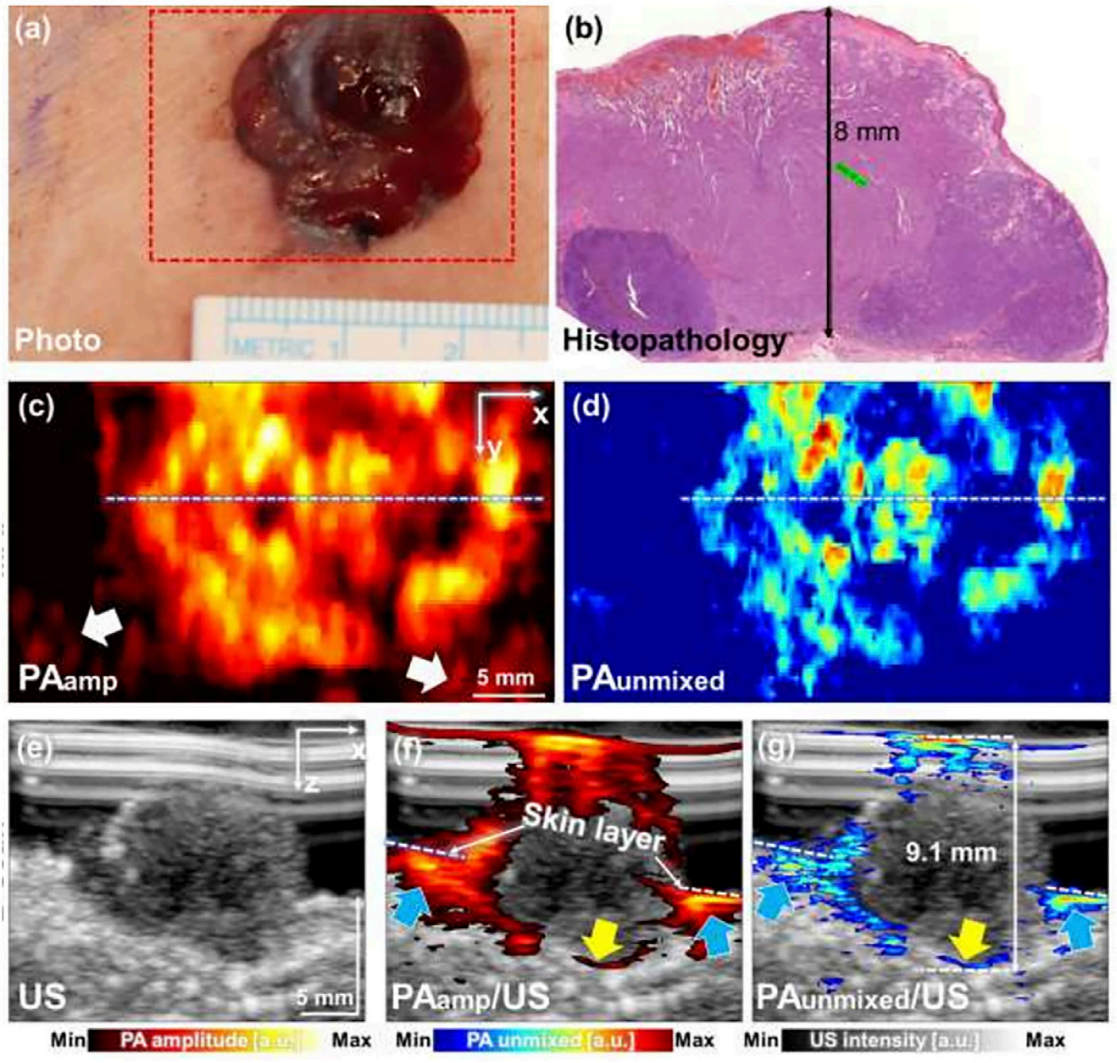
Combined PAT/US imaging of skin cancers. PAT image of a skin melanoma acquired simultaneously with a US image. **(A)** Clinical photo and **(B)** histopathology of melanoma on human chest, **(C)** PAamp MAP and **(B)** PAunmixed MAP images along the area of red dashed box in **(A)**; **(E)** US image of melanoma; **(F)** PAamp/US image and **(G)** PAunmixed/US image along the white dashed lines in **(C,D)**, respectively. PAT: photoacoustic tomography; US: ultrasound; PAamp, photoacoustic amplitude; PAunmixed, photoacoustic unmixed melanoma; MAP, maximum amplitude projection. Reprinted with permission from Park et al. (2021).

Detection and quantification of melanoma depth have been reported with several types of PAT/US imaging systems *in vivo* (Li M et al., 2018; Wang S. L et al., 2021; Park et al., 2021). This unique PAT/US imaging here opens unprecedented capabilities for high-resolution skin imaging at scalable depths *in vivo*. A pilot study showed six melanoma patients examined *in vivo* using the 3D MSOT imaging system (Figure 5). By using a MSOT/US system, melanoma of various sizes, locations (chest, thigh, heel, feet, and palm) and forms (1.3–30 mm lateral diameter, 0.6–9.1 mm depth) were detected by US technology. Feeding vessels were visualized in the melanoma using 3D PAT image, suggesting the neovascularization in the tumor. An analysis of those MSOT data confirmed a high correlation between the depth of the melanoma and its histology (Park et al., 2021). Thus, this PAT(MSOT)/US system, in particular with 3D reconstruction, will possibly serve as an important noninvasive imaging tool in determining the stage of skin cancer, in deciding the excision region of the cancer in surgery. Furthermore, it would improve the prognosis of the skin melanoma patients in near future. However, these works were still preliminary attempt for the diagnosis. In order to further explore the potential of PAT/US in early diagnosis of melanoma, more experiments *in vivo* are necessary with different subtypes and stages of melanoma. Further, deep learning (DL) and artificial intelligence (AI) algorithms can be combined to detect the invasion depth and boundary of melanoma more precisely.

### Extremities imaging

Monitoring the microcirculation in target tissue is crucial in assessing bone diseases, inflammation of the synovium and peripheral vascular diseases, such as diabetic foot, synovitis, rheumatoid arthritis, and arterial embolization in lower extremity. However, conventional imaging modalities are focused on diagnosis in major arteries, and are limited to provide microvascular information in early stages of the disease. PAT imaging reliably quantify vascular parameters noninvasively in human extremities (Kruizinga et al., 2013; Xu et al., 2013; Jo et al., 2017; van den Berg et al., 2017; Choi et al., 2022). However, the localization of the PAT signal may require another modality. A unique advantage of US is its good localization, non-ionizing nature, and ability to penetrate soft tissues. Given the abovementioned advantages of PAT and US technique, PAT/US image system has potential for clinical extremities health assessment.

To facilitate clinical use, a costumed 3D PAT/US system has been conducted for finger imaging in a healthy human *in vivo* (Oeri et al., 2017). Tomography consists of four separate and fully automated removable curved sensors that can image all three finger joints. This study has provided new opportunities in finger diagnostics. However, noises (similar frequency as signals) were observed in raw data of some arcs, yielding streaking artifacts in reconstructed images in certain regions.

Recently, researchers challenged the use of US-guided PAT to visualize human bones (Feng et al., 2020). The results suggested that it can distinguish PAT signal of human cortical and trabecular bones *in vivo*, as well as the surrounding soft tissue. However, this work has not provided a quantitative assessment based on trabecular bone PAT signal. And then, MSOT/US was applied for assessment of 17 systemic sclerosis (SSc) patients (5 out of 17 was in early phase) with nailfold damage, 5 primary Raynaud’s phenomenon (PRP) and 9 health controls (Daoudi et al., 2021). Since MOST can quantitatively evaluate capillary density and hemoglobin (Hb) contents of the third fingers and US is capable of measure skin thickness of the lesions, this hybrid method could help to distinguish early SSc from PRP individuals and health controls in both Hb contents and skin thickness.

**FIGURE 6:**
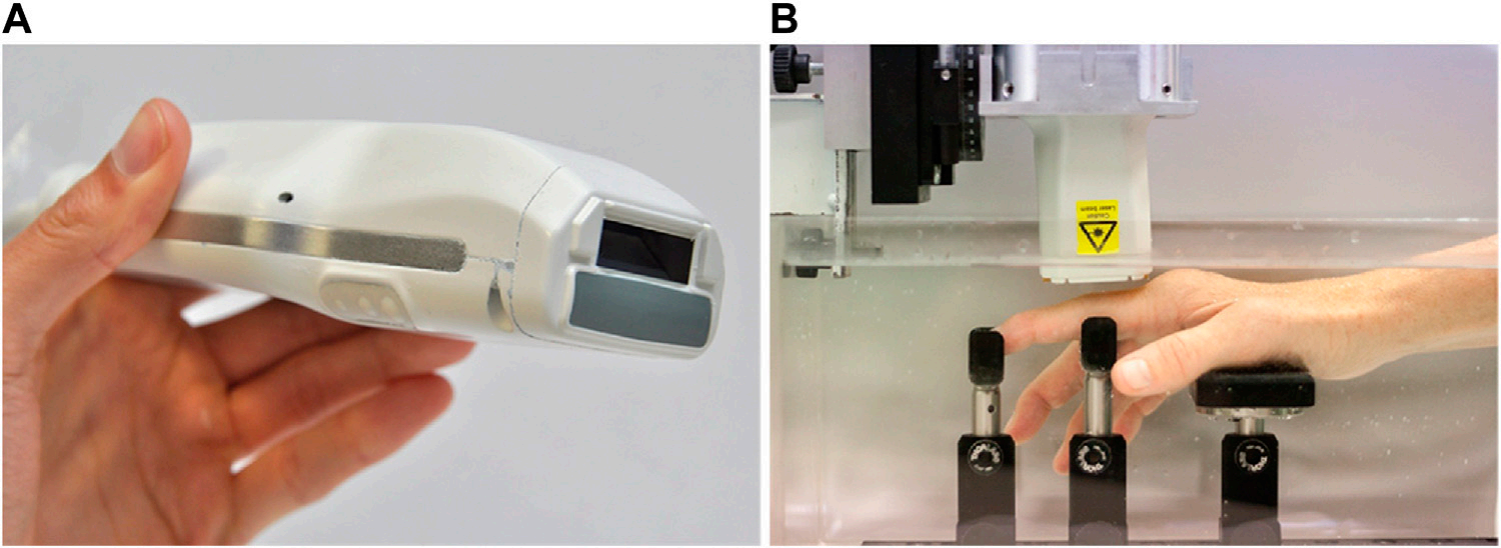
**(A)** A PAT/US probe with a front-end view showing the light delivery window (dark aperture) and gray acoustic lens in medium; **(B)** The patient’s hand is submerged in water and is supported by a series of braces. The sensor is mounted on a motorized 2-axis stage and positioned above the joint. PAT: photoacoustic tomography; US: ultrasound. Reprinted with permission from [van den Berg P. J, Daoudi K., Bernelot Moens H. J., and Steenbergen W. (2017). Feasibility of photoacoustic/ultrasound imaging of synovitis in finger joints using a point-of-care system. Photoacoustics. 8,8–14. doi: 10.1016/j.pacs.2017.08.002].

**FIGURE 7:**
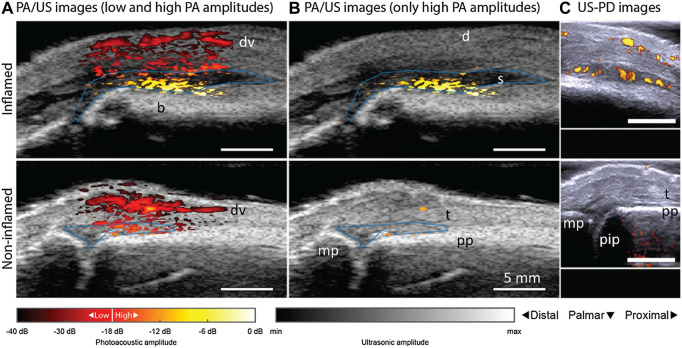
PAT/US and US/PD images of an inflamed contra-lateral (bottom row) and non-inflamed (upper row) joint from a patient with rheumatoid arthritis. PAT/US images in **(A)** show a difference in color between inflamed and non-inflamed corresponding to an increase in amplitude levels. If low PAT amplitudes are discarded in **(B)**, only features in the inflamed joint are seen. Figure **(C)** shows the corresponding US-PD images. Blue lines in PAT/US images indicate ROIs used for PAT feature quantification in synovial space. The 0 dB level is the maximum PAT amplitude produced by an inflamed joint. d, dermis; dv, dorsal vein; pp, proximal phalanx; pip, proximally located interphalangeal joint; mp, midline phalanxes; s, synovium; t, extensor tendon; PAT, photoacoustic tomography; US, ultrasound; PD, powered Doppler. Reprinted with permission from [van den Berg P. J., Daoudi K., Bernelot Moens H. J., and Steenbergen W. (2017). Feasibility of photoacoustic/ultrasound imaging of synovitis in finger joints using a point-of-care system. Photoacoustics. 8,8–14. doi: 10.1016/j.pacs.2017.08.002.

Furthermore, with the increase need of fast diagnosis devices, POC technique has gained popularity and also been studied for possible application in clinical settings. The feasibility of a portable PAT/US system was evaluated for clinically evident synovitis (van den Berg et al., 2017). The proximal interphalangeal joints of the inflamed and non-inflamed joints of ten patients were examined and compared with the joints of 7 healthy volunteers. PAT scan, power Doppler US (US-PD) were performed (Figure 6). The PAT probe in this study is sensitive to vessels and vascular networks of 0.2 mm in size. PAT signals in inflamed joints increased significantly, compared with contralateral non-inflamed joints and with joints from healthy volunteers, which was highly correlated with US-PD (Figure 7). Therefore, combined PAT with US using a compact handheld probe is able to detect clinically manifest synovitis. However, one of the technical limitations of this system was the lack of shared access to high-quality PAT and US images. The short delay between the two may have contributed to inaccuracy caused by the unexpected movement of the fingers. The restriction on this setting will be addressed in future versions, resulting in almost simultaneous access to PAT and US images.

Future applications of PAT/US system in extremities can take advantage of its multi-spectral imaging capabilities, allowing the estimation of the SO_2_ of lesion tissues. In next, quantitative parameters acquired from MOST on different bone mineral densities of long bones will be available. The bone and joint evaluation platform is needed to provide both microstructural and metabolic information, which is highly valuable for diagnosis and grading as well as monitoring of osteoporosis therapy and other osteoarticular diseases. Furthermore, targeted PAT/US contrast agents based on molecular markers need to be investigated, providing information about inflammation similar to positron emission tomography.

### Large blood vessels imaging

**FIGURE 8:**
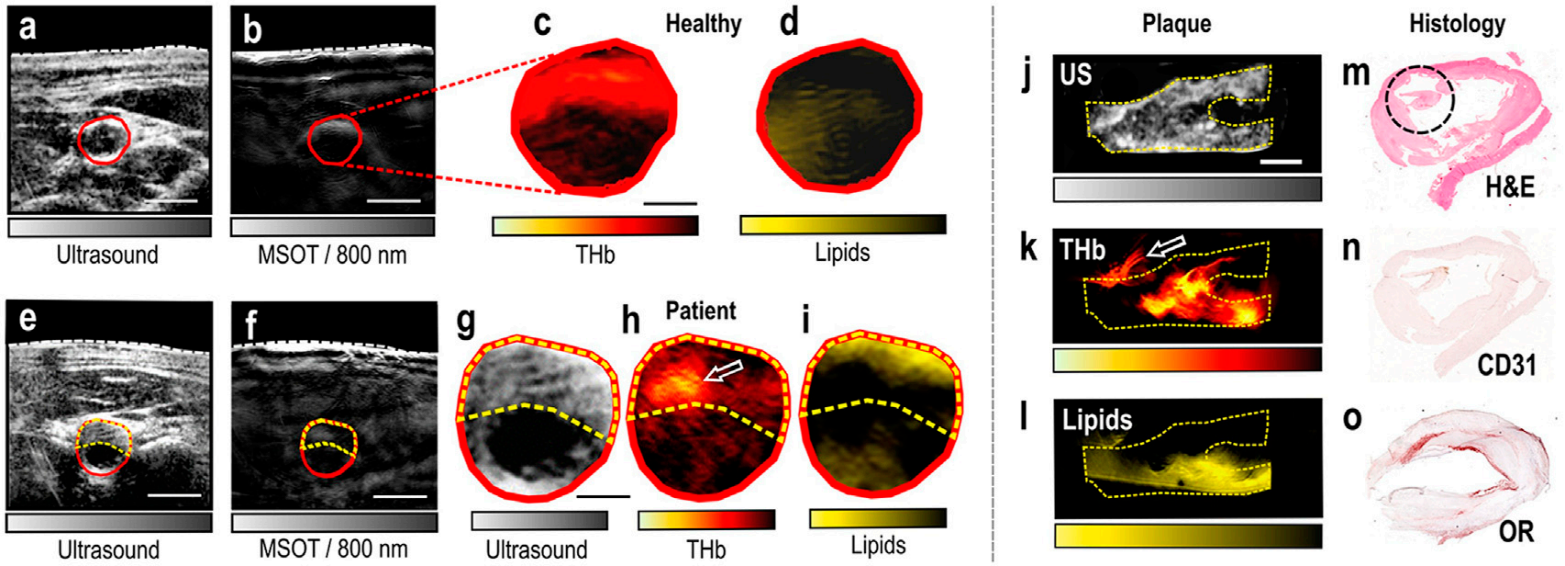
MSOT/US imaging of the carotid artery of a healthy volunteer and a patient, MSOT/US imaging and histology of an excised plaque. **(A)** US image of the transverse section of the carotid artery of a healthy volunteer. **(B)** Corresponding MSOT image at 800 nm. White dashed line: skin surface. The carotid lumen area is demarcated in red. Scale bars: 5 mm. **(C)** Magnification of the lumen area of the spectrally unmixed MSOT image in **(B)** showing the HbT signal. **(D)** Same magnification showing the Lipids signal. Scale bar: 2.5 mm. **(E)** US image of a patient with carotid atherosclerosis. **(F)** Corresponding MSOT image at 800 nm. White dashed line: skin surface. The lumen is demarcated in red and the plaque area with a yellow dashed line. Scale bars: 5 mm. **(G)** Magnification of the arterial cross-section in US. **(H)** Magnification of the arterial cross-section of the spectrally unmixed MSOT image in **(F)**, showing the HbT signal. White arrow: region of increased HbT content. **(I)** Same magnification showing the Lipids-signal. Scale bar: 2.5 mm. **(J)** Sagittal view of the excised plaque in US. **(K)** Same view of the plaque in corresponding unmixed HbT image. White arrow: postoperatively attached suture. **(L)** Same view of the plaque in unmixed MSOT Lipids image. Scale bar: 4 mm. **(M)** Histological view with H&E staining. The black circle shows a region with thrombotic and erythrocytes components. **(N)** Histological view with CD31-staining targeting the neovascularization. **(O)** Histological view with OR-staining showing the lipid content of the plaque. MSOT: multispectral optoacoustic tomography; US: ultrasound. Reprinted with permission from [Karlas A., Kallmayer M., Bariotakis M., Fasoula N. A., Liapis E., Hyafil F., et al. (2021). Multispectral optoacoustic tomography of lipid and Hb contrast in human carotid atherosclerosis. Photoacoustics.23,100283. doi: 10.1016/j.pacs.2021.100283].

Lipids in plaques are an important marker of atherosclerosis. Among endogenous contrast compositions, lipid is one of the most commonly used PAT biomarkers and has been intensively studied. An intravascular PAT/US probe was applied to visualize blood lipid in arteries, which was able to successfully detect and distinguish plaque lipids in human coronary arteries from adventitial fat *ex vivo* (Wu et al., 2015). This imaging technique has demonstrated its ability to identify features of plaque instability but come with limitations, such as the use of contrast agents, long examination times and poor portability. Recently, five patients with carotid atherosclerosis, five healthy volunteers and two excised plaques, were scanned with handheld MSOT noninvasively (Karlas et al., 2021a). Spectral unmixing allowed visualization of lipid and Hb content within three ROIs: whole arterial cross-section, plaque and arterial lumen. This finding introduces MSOT as a new tool for molecular imaging of human carotid atherosclerosis and opens new opportunities for research and clinical evaluation of carotenoid plaques. Following this, MSOT, US and colored Doppler imaging of the carotid arteries in healthy individuals was performed, along with blood flow and oxygenation measurements. (Wu Y et al., 2021). This work demonstrates that multimodality has the potential to provide comprehensive information with increasing accuracy (Figure 8). However, lipids generated just moderate PAT signals at wavelength of 1734 nm, making PAT images at this wavelength vulnerable to noise. To tackle this problem, noise reduction and probe sensitivity will improve accuracy and reliability of lipid identification.

### Lymph system

**FIGURE 9:**
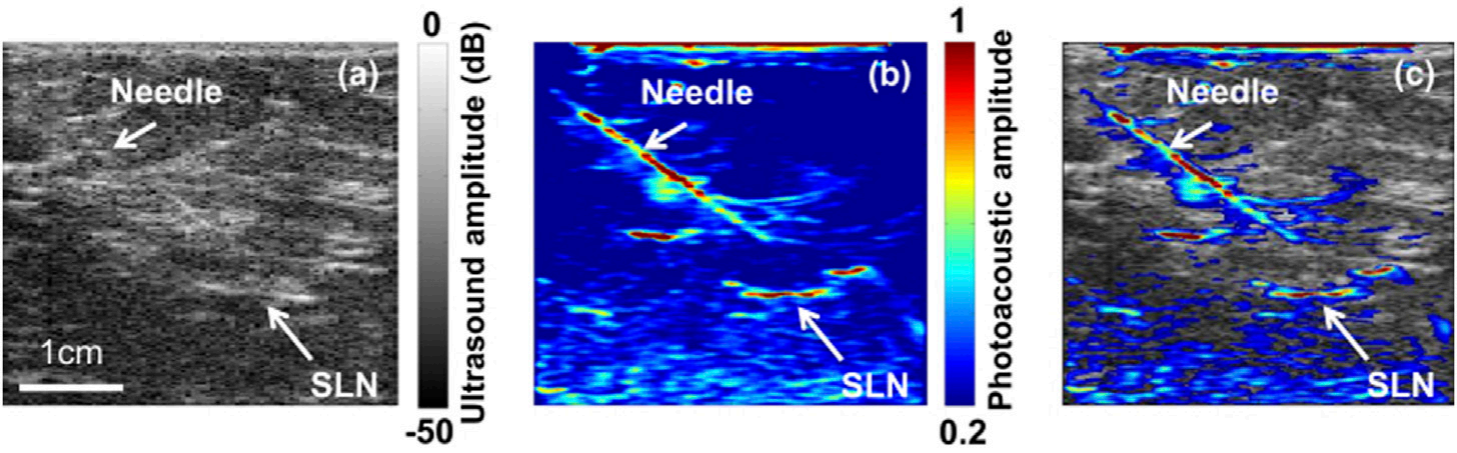
Images of the SLN and the needle acquired using PAT/US *in vivo*. **(A)** US image showing the lymph node and needle *in vivo*. **(B)** PAT image of the SLN and needle *in vivo*. **(C)** Coregistered PAT/US image of the SLN (long white arrow) and needle (short white arrow). SLN: sentinel lymph node; PAT: photoacoustic tomography; US: ultrasound. Reprinted with permission from [Garcia-Uribe A., et al. (2015). Dual-Modality Photoacoustic and Ultrasound Imaging System for Noninvasive Sentinel Lymph Node Detection in Patients with Breast Cancer. Sci Rep.5, 15748. doi: 10.1038/srep15748].

Detecting regional lymph node metastasis is important in cancer staging, as it guides patient prognosis and treatment strategy. Sentinel lymph node biopsy (SLNB) heroine is an accurate and less invasive alternative to axillary lymph node dissection (Aragon et al., 2022). Since high sensitivity to dyes, high spatial, contrast and temporal resolution, enough imaging depth are key requirements for SLNB (Song et al., 2008; Kim and Chang, 2018), blue dye is an ideal contrast agent for PAT due to its strong optical absorption. Accurate identification of sentinel lymph node (SLN) by PAT/US can enable SLN sampling using fine needle aspiration biopsy (FNAB) for a minimally invasive approach to axillary staging (Garcia-Uribe et al., 2015). As a non-ionizing hybrid imaging method, coregistered PAT/US imaging can detect SLNs and lymphatic vessels using methylene blue dye (Figure 9). However, 5 fps of coregistered images is not sufficient for real-time biopsy guided by PAT/US in clinical applications. To achieve a higher frame rate of reconstructed PAT/US images, DAQ computer is necessary to be improved, aiming for real-time visualization in preoperative evaluation in patients with newly diagnosed invasive breast cancer in the future.

### Molecular imaging

The biomedical PAT is based on the absorption spectra of intrinsic absorbers present in human tissue, such as HbO_2_ and HbR (Laufer et al., 2005; Pan et al., 2010), lipids (Guggenheim et al., 2015; Kole et al., 2019), melanin (Kratkiewicz et al., 2019), water (Xu et al., 2011), RNA and DNA (Yao et al., 2010). When these intrinsic chromophores are not sufficient to reveal the disease, extrinsic contrast agents can be utilized to target different diseased biomarkers to increase molecular sensitivity and specificity. Applications of PAT imaging in the clinical research have shown promising results with endogenous contrast (Wu et al., 2014; Wu et al., 2019b), including in inflammatory bowl (Knieling et al., 2017), dermatology (Masthoff et al., 2018) and breast cancer (Nyayapathi and Xia, 2019). For US imaging, Optison (human serum albumin stabilized perflutren microspheres), Definity (perflutren lipid microspheres), SonoVue (phospholipid-stabilized microbubble), and Sonazoid (F- butane encapsulated in a lipid shell) have been approved for clinical use by the FDA (Cohen et al., 1998; Morel et al., 2000; Halpern et al., 2002; Datta et al., 2008).

Dual-modal contrast agents for PAT/US imaging have been studied to improve the diagnostic sensitivity and specificity. In order to enable molecular PAT/US detection, contrast agents can be specifically tailored to molecular targets of relevance to tumor metastasis, including those biomarkers expressed during lymphangiogenesis, as well as those expressed by tumors. Studies on exogenous contrast agents for PAT/US imaging biomedical application have been reported (Kim et al., 2010a; Xu et al., 2010; Jeon and Kim, 2014; Paproski et al., 2016; Zhang et al., 2016; Bayer et al., 2017; Park et al., 2017b; Chitgupi and Lovell, 2018; Shi et al., 2021). Angiogenesis, the formation of new blood vessels, is a hallmark of many diseases, including tumor and inflammation. In response, those lesions can be detected by PAT based on Hb, which is one of the major chromophores. The treatment of the bubble surface to target specific molecules can broaden the applications of these contrast agents using combined PAT and US imaging. Current contrast agents for PAT/US dual-modality include the conjugating NBs with cancer-targeting ligands (Xu et al., 2010), Texas Red dye in poly (lactic-co-glycolic acid) (PLGA) NBs (Kim et al., 2010c) and gold nanotracers (Au NTs) labeled with mesenchymal stem cells (MSCs) for monitoring disease processing (Nam et al., 2012), encapsulated gold nanorod human serum albumin (HSA)-shelled microbubbles (AuNR-HSA) for thermotherapy (Wang et al., 2012), liquid perfluorocarbon (PFC) nanodroplets with encapsulated plasmonic nanoparticles and encapsulated-ink microbubbles for biomedical application (Jeon M., and Kim, C., 2014). As mentioned above, most of contrast agents are applicable in preclinical trails, only few of them have potential opportunities towards clinical translation, such as particular microbubbles loaded with ICG. In addition, by targeting specific molecules on the bubble surface, these contrast agents can be used for simultaneous PAT and US imaging, including tumor borders, intracranial imaging, and molecular imaging of primary and metastatic tumors in the future.

### Other clinical applications

In the last few decades, through intensive study and elaboration, PAT/US dual-modal technology can therefore visualize the human structures or tissues extending to whole-body from the organelles to organ range, including prostate *in vivo* (Kothapalli et al., 2019; Agrawal et al., 2020), placenta *ex vivo* (Xia et al., 2019; Maneas et al., 2020), bowels *in vivo* and *ex vivo* (Knieling et al., 2017; Leng et al., 2018 Yang et al., 2019), periodontal health *in vivo* (Mozaffarzadeh et al., 2021) and spine in human cadaver (Gonzalez et al., 2021).

In prostate and some of bowels researches, intracavity PAT/US probes were conducted *in vivo*. In order to image the entire prostate and circumferential bowel wall, the FOV probes should be designed as large as possible, which in the range of 130°–150° at least (Agrawal et al., 2020). However, compared to the conventional clinical PAT/US devices, these PAT/US probes were lack of sufficient elements within the transducers and high performance DAQ systems. On the other hand, the probe should be small enough to relieve the patient’s pain during dectetion. Accordingly, these two restrictions limit the spatial and contrast resolutions of intracavity images. The advanced PAT/US system with higher performance intracavity probe could solve this issue, due to its clear visualization of microvasculature distribution in prostate (Kothapalli et al., 2019; Agrawal et al., 2020) and bowel diseases (Knieling et al., 2017; Leng et al., 2018; Yang et al., 2019). Furthermore, in order to achieve better clinical results in the future, a wider FOV of multi-modal probe with a 360° visualization, a better resolution of the system and 3D image reconstruction are needed in the second generation PAT/US device. Meanwhile, further study on the large cohort, multi-center datasets of this approach is needed in monitoring clinical efficacy.

To investigate the ability of PAT/US to image deeper tissue, research groups have performed PAT/US imaging on *ex vivo* human placentas (Xia et al., 2019; Maneas et al., 2020). It is suggested that PAT imaging combined with US tracking could provide a useful method for detecting the placental vasculature during minimally invasive fetal surgery (Xia et al., 2019). Moreover, the 3D dual-modal PAT/US imaging appears to be promising for visualizing human placental vasculature in healthy and twin-to-twin transfusion syndrome (TTTS) treated placentas (Maneas et al., 2020). However, limitations of proposed method include insufficient sensitivity in detecting vessels at all depths in the placenta, and poor spatial resolution in detecting the smallest vessels. It is one of the key challenges for the application in future studies. Further MSOT imaging could be used to discriminate between coagulated and non-coagulated blood based on their different absorption spectral during the treatment of TTTS.

Another challenge application in deep tissue is utilized for a human cadaver vertebra imaging by PAT/US guidance system. This combined system was promising to assist surgeons with identifying and avoiding impending bone breaches during pedicle cannulation in spinal fusion surgeries (Gonzalez et al., 2021). However, the research is still conducted on human cadavers. Extensive preclinical trials are needed before it can be used in humans *in vivo*.

A pilot study of simultaneous visualization of the teeth and periodontium is of significant clinical interest for image-based monitoring of periodontal health (Mozaffarzadeh et al., 2021). It was found that a successful visualization periodontal anatomy and periodontal pocket depths in humans using a dual-modal PAT/US imaging system for the first time. This work demonstrated that 3D PAT/US images allow for accurate measurement and visualization of periodontal features, including the periodontal anatomy, enamel pigmentation, and pocket depth. Efforts are made to remove shaking artifacts from 3D PAT/US images by a specific algorithm. In spite of this, the calculation is complicated and needs to be improved in the future work.

## Summary and future perspectives

In this review, we focus on the application and advances in dual-modal PAT/US imaging technology in clinical translations and discussed in details in a systematic way. We explained the principle of PAT/US dual-modality and also discussed a variety of existing PAT/US systems and summarized their key characteristics in Table 1. In the next, we detail clinical applications of current PAT/US system. Compared to traditional PAT imaging, PAT/US detection owns several advantages in clinical applications, such as optical transparency, material flexibility and anti-electromagnetic interference. Several advanced PAT/US imaging technologies have taken another step forward for clinical translation. However, further improvements are needed to make it more clinically compatible.

**TABLE 1:** Comparison of various configurations for clinical PAT/US dual-modal systems.

Transducers	f_0_ (MHz)	No. of elements	FOV	Organs/Tissues	References
Intravascular, full-ring array	20–50	Single	360°	coronary atherosclerosis, *ex vivo*	Karpiouk et al. (2012)
Intravascular, full-ring array	45	—	360°	Coronary artery, *ex vivo*	Wu et al. (2014)
Handheld duplex probe	—	—	—	Breast, *in vivo*	Neuschler et al. (2017)
Handheld, linear array	3	256	125°	Breast, *in vivo*	Becker et al. (2018)
Handheld, linear array	0.1–12	128	—	Breast, *in vivo*	Oraevsky et al. (2018)
Rotate, concave array	10	384	360°	Breast, *in vivo*	Kelly et al. (2020)
Handheld, linear array	5.8	192	3D	Breast, *in vivo*	Yang et al. (2020)
Handheld, linear array	5	256	125°	Breast, *ex vivo*	Goh et al. (2019)
Handheld, linear array	5–12	256	—	Sentinel lymph node, *in vivo*	Garcia-Uribe et al. (2015)
Handheld, curved array	∼7.5	64	172°	Thyroid, *in vivo*	Dima and Ntziachristos, (2016)
Handheld, linear array	5.8	192	—	Thyroid, *in vivo*	Yang et al. (2017)
Handheld, linear array	8.5	128	—	Thyroid, *in vivo*	Kim et al. (2021)
Transrectal, linear array	5	64	± 20°	Prostate, *in vivo*	Kothapalli et al. (2019)
Transrectal, linear array	4–8	128	135°	Prostate, *in vivo*	Agrawal et al. (2020)
Handheld, linear array, EC-12R, Alpinion	—	—	—	Melanomas, *in vivo*	Park et al. (2021)
Handheld, linear array,CL15-7, Philips	8.9	—	180°	Joint, *in vivo*	Jo et al. (2017)
Fixed, full-ring	5	512	360°	Fingers, *in vivo*	Xia et al. (2015)
Handheld, spherical array	4	256	90°	Fingers, *in vivo*	Xu et al. (2013)
Handheld, linear probe	7.5	128	∼30°	Fingers, *in vivo*	Daoudi et al. (2021)
Fixed, curvilinear array	4–5	64–512	135°–270°	Fingers, *in vivo*	Mercep et al. (2018)
Rotated	3.5	2	∼10°	Fingers, *in vivo*	Liu and Zhang, (2016)
Fixed, linear array	14	128	—	Fingers, *in vivo*	van den Berg et al. (2017)
Fixed, arc-like array	10	768	360°	Fingers, *in vivo*	Oeri et al. (2017)
Handheld, linear array	3–12	128	—	Forearm, *in vivo*	Kim et al. (2016)
Fixed	0.5	—	—	Bone, *in vivo*	Feng et al. (2020)
Handheld, linear array	—	—	—	Bowel, *in vivo*	Knieling et al. (2017)
Endorectal probe	20	—	360°	Bowel, *in vivo*	Leng et al. (2018)
Endorectal probe	6–10	—	—	Bowel, *in vivo*	Yang et al. (2019)
Handheld, linear array	10, 20.5, and 40	128	4.5 × 3.5 cm	Periodontal, *in vivo*	Mozaffarzadeh et al. (2021)
Handheld, linear array, EC-12R, Alpinion	4	128	—	Spinal pedicle, *ex vivo*	Gonzalez et al. (2021)
Handheld, linear array, SonixMDP	10	128	62°	Placenta, *in vivo*	Xia et al. (2019)
Handheld, linear array, AcousticX	9	128	3D	Placenta, *ex vivo*	Maneas et al. (2020)

PAT, photoacoustic tomography; US, ultrasound; FOV, field of view; 3D, three-dimensional; --, not mentioned.

Future work of this hybrid system may include: 1) Real-time 3D reconstruction technology would be a good future for advanced PAT/US system, which can produce a stereo vision of lesions for radiologists and clinicians. Thus, PAT/US imaging systems with 3D imaging require new methods and materials for systematic testing which can help in decision-making during clinical translation; Furthermore, 3D printed biomodel for simulation of anatomic angioarchitecture of lesions will facilitate surgeon to develop and evaluate a surgical protocol; 2) An advanced PAT/US imaging reconstruction algorithm need to be further proposed based on the previous studies (Jiang, 2015; Wang et al., 2022) to improve lateral resolution of hybrid image, as well as temporal resolution aiming to simultaneously displaying structural, functional, and molecular information; 3) The miniaturization of a PAT/US dual-modal system with significant improvements in the performance of portable laser pump sources, high performance DAQ computers and handhold transducer with smaller size and lighter weight; In next, POC would refer to PAT/US examination outside the lab, such as bedside care, in emergency departments, surgery monitoring or ambulant first aid. It will be a widely used tool for imaging and therefore reducing the time in clinical decision making, emergency and medical education in the coming future. 4) To explore other human organs researches and translations, such as the lungs, pediatric heart, fetus, uterine, neck organs, and others. Due to the dependent optical attenuation depth and wavelength and unknown optical and acoustic heterogeneities limit PAT/US imaging performance in deep tissue regions; therefore, efficient deep tissue’s energy transfer system should be developed in next work; 5) PAT/US dual-modal exogenous contrast agents, such as particular microspheres loaded with ICG, have potential opportunities in future to monitor tumor process, metastasis in different part of human body; 6) The multifunctional nanocomposites with dual-modal PAT/US imaging and synergistic therapy will have great application value in different clinical fields involving tumor, vascular plaque, antimicrobial therapy and others in the coming future; 7) PAT/US dual-modal system integrated with AI applications have a broad research prospect in the diagnosis and treatment of human diseases. The previous simulation platform has the potential to generate large-scale application-specific training and test datasets for AI, enhancing AI assisted PAT/US imaging (Agrawal et al., 2019; Hariri et al., 2020; Agrawal et al., 2021b). DL would substantially impact the advancement of modern PAT/US imaging processing methods. Future scope of this work involves 3D simulations and validation studies of different organs to simulate real optical and acoustic heterogeneity, artifacts, shadow effects, and systemic noise. Thus, AI algorithms can be combined to detect the invasion depth and boundary of tumors more precisely in the coming future; 8) At last, FDA cleared PAT/US devices have a greater potential to be a quicker way for clinical application and translation.

We hope this review can be helpful for researchers who wish to learn more about PAT/US dual-modal detections and to use PAT/US dual-modal imaging in their clinical applications.
